# Reuse of Explanted Pacemakers: An Option for Economically Underprivileged Patients in Developing Countries

**Published:** 2007-10-22

**Authors:** Johnson Francis, R Anilkumar, Harry Mond

**Affiliations:** 1 Professor of Cardiology, Calicut Medical College, Kerala, India; 2 Specialist cardiologist, Belhoul Speciality Hospital, Dubai; 3 Associate Professor of Medicine, University of Melbourne and Monash University, Melbourne, Australia

**Keywords:** pacemaker, reuse

Permanent pacemaker implantation is an established mode of life saving therapy for patients with symptomatic bradycardia. As the human population is aging world-over, the need for pacemakers are also increasing. Reviewing the results of the 2001 World Survey on Cardiac Pacing and Implantable Cardioverter Defibrillators (Table 1), it is evident that not all patients who require pacemakers are receiving them, particularly in the Asia-Pacific region. The largest implanting country per head of population is Germany, although the largest numbers implanted are in the United States of America. In contrast, the countries of Asia have much smaller numbers with Myanmar having less than one implant per million population per year [1].

There are many reasons for the low pacemaker implant rates in Asia, although economic factors are by far the most important. The cost of a basic single chamber pacemaker is above $US1000. This is often more than the annual income of the average citizen in many countries. Therefore, many individuals, particularly those who would otherwise yield productive lives with an implanted pacemaker, are currently being denied this opportunity due to economic constraints. Local physicians, hospital administrators and health bureaucrats are very aware of the need to get more pacemakers within reach of the needy and pacemaker manufacturers are now producing much cheaper basic models for exclusive Third World use. However, despite this, the pacemaker hardware and implanting costs per patient remain excessive and well beyond the financial range of both patients and medical charities.

With improved pacemaker power source longevity, it has now become increasingly apparent, that many normally functioning long-life pulse generators are outliving their recipients. The result is that many pulse generators with a substantial remaining power source have been removed from deceased patients and with appropriate refurbishing are available for reimplantation. The concept of pulse generator refurbishing is not new and has been widely and safely performed since the dawn of pacemaker technology [2-10]. The explanted pulse generators are cleaned, tested for remaining power source life and functional integrity by strict protocols and then resterilized [5]. Ironically most of the reports of pulse generator refurbishment have not come from third world countries where the implants have been in the needy, but rather from European, North American and Australian sites, where free public health systems have been available [3,5,6,9,10]. Because of the difficulty removing and resterilizing previously implanted pacing leads, such refurbishment has never been reported and unlikely to have been performed. Consequently any refurbished pulse generator would require new pacing leads at the time of an initial implant.

There are several concerns with the reuse of pulse generators. Fully informed consent should be taken both from donor relatives and recipients. Use of explanted pacemakers should be considered only if the patient cannot afford a new pulse generator. Companies will obviously not extend the warranty for the refurbished product. Hence reuse should be only considered when the cost of implantation including that of the new lead is significantly lower than that of the new pulse generator. The refurbishing process has a strict protocol and for obvious legal and commercial reasons, pacemaker companies will not undertake the responsibility nor provide a warranty on the work performed. There are no commercial companies prepared to undertake such a task in a legal minefield and if so the costs may well be prohibitive. A minor problem easily overcome with programming is pulse generator-lead compatibility. For example, a high polarization lead used with an automatic capture detection system which utilizes the evoked response can theoretically lead to inhibition of the pacemaker.

Any attempt to undertake a pulse generator refurbishing program requires Governmental and legal approval [2] and the whole process requires strict supervision. Not surprisingly, studies on pacemaker reuse have not shown a higher incidence of infections in refurbished pacemakers vs new implants [2-5]. Washing with distilled water under sterile conditions and gas sterilization with ethylene oxide is a recommended method of processing the explanted generators [5].

Although it may appear impossible to undertake an economic active pulse generator refurbishing program today, it should be noted that the large study by Balachander et al [8] from India was the result of collaborative efforts of a French voluntary organization (STIMUBANK) and JIPMER in Pondicherry. India. STIMUBANK is based at Nancy, France, and collects explanted pacemakers as well as shelf-expired pacemakers and ships them for needy patients in developing countries. JIPMER has followed up more than 1000 patients implanted with refurbished pacemakers over the past twenty years and observed results comparable with new implants regarding longevity and complications (Personal communication  R Anil Kumar)

In summary, therefore, refurbishing of pulse generators is safe and technically feasible. The Governmental and legal hurdles are not insurmountable, provided there is a strict refurbishing protocol under proper supervision. Care should be taken to avoid costly commercial exploitation at any level of the enterprise.

## Figures and Tables

**Table 1:**
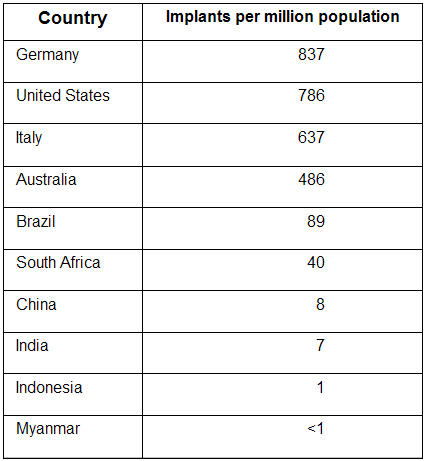
Number of pacemakers Implanted in Selected Countries (2001)

Data from Mond et al [1]
